# Supervisor Feedback and Innovative Work Behavior: The Mediating Roles of Trust in Supervisor and Affective Commitment

**DOI:** 10.3389/fpsyg.2020.559160

**Published:** 2020-09-11

**Authors:** HyeonUk Bak

**Affiliations:** Korea Research Institute for Local Administration, University of Seoul, Seoul, South Korea

**Keywords:** affective commitment, supervisor feedback, innovative work behavior, trust in supervisor, local government, public servant, South Korea

## Abstract

This study aimed to explore how the mechanisms of supervisor feedback affect innovative work behavior (IWB) among local government employees and to examine the mediating roles of trust in supervisor and affective commitment, using organizational support theory (OST) and social exchange theory. The results from a cross-sectional study based on a sample of 1,699 local government employees from 65 local governments indicated that supervisor feedback had a direct effect on IWB. Trust in supervisor and affective commitment significantly mediated the relationship between supervisor feedback and IWB. The findings of this study contribute to an advanced understanding of the supervisor feedback-IWB relationship by testing the mediation model in the local government context.

## Introduction

Recently, there has been a growing interest in innovative work behavior (IWB) among both scholars and practitioners (Bysted and Jespersen, 2014; Bysted and Hansen, 2015; Günzel-Jensen et al., 2018; Miao et al., 2018; Hansen and Pihl-Thingvad, 2019; Khan and Khan, 2019). IWB refers to employee’s behaviors to improve individual and/or organizational work outcomes by generating, promoting, implementing, and realizing new ideas and is regarded as a vital factor for organizational performance improvement and sustainable development (Scott and Bruce, 1994; Janssen, 2000; Shanker et al., 2017; Hansen and Pihl-Thingvad, 2019; Saether, 2019). In addition, employees improve their individual performance through IWB (e.g., fixing errors in service delivery, learning from processes for identifying and correcting the errors, and generating creative ideas for work processes; Fernandez and Moldogaziev, 2013; Günzel-Jensen et al., 2018). Researchers also contend that IWB contributes to the improvement of service delivery and problem-solving abilities (De Vries et al., 2016).

Supervisor feedback is crucial in every organization because it is important in communication between supervisors and subordinates. It has been known that supervisor feedback plays a role in changing their subordinates’ attitudes and behaviors. Previous studies have indicated that employees who receive performance or developmental feedback from their supervisors are likely to display positive behavioral and attitudinal outcomes, such as organizational citizenship behavior (OCB; Peng and Chiu, 2010), organizational commitment (OC; Eisenberger and Stinglhamber, 2011), trust (Nyhan, 2000), job satisfaction (Jong, 2016), and performance improvement (Favero et al., 2014). However, relatively little empirical research has examined the role of supervisor feedback on IWB, and its effects on IWB is veiled. This is rather surprising, given that job resources, such as performance or developmental feedback, are closely related to employees’ positive attitudes and behaviors (Zhou, 2003; Favero et al., 2014). Thus, there is a need to clarify how individual and organizational factors influence IWB.

This study makes several key contributions. First, this study is to test a mechanism that explains the relationship between supervisor feedback and IWB. Considering the call for a better understanding of the role of job resources as important antecedents of IWB, this study explores the role of job resources (i.e., performance/developmental feedback) as an individual motivational factor of the extra-role behavior, IWB. Given the increasing importance and interest in feedback and IWB in the workplace, exploring the mechanisms of supervisor feedback and IWB may enrich the feedback, job resources, and IWB literature. It also may provide practical insight for organizational leaders, supervisors, and human resource managers in terms of effective feedback delivery and spread of IWB in the organization.

In keeping with the call for empirical testing of rarely explored mediators of the motivational job resources-IWB relationship (Li and Hsu, 2016), two key mediators, trust in supervisors and affective commitment, are included to help understand the underlying mechanisms through which supervisor feedback influences IWB in the workplace. Drawing from organizational support theory (OST) and social exchange theory, this study attempts to fill the research gap and develop the unique framework that examines the mediating roles of trust in supervisor and affective commitment in linking supervisor feedback and IWB in the public sector.

Last, this study makes theoretical and practical contributions about the relationship between IWB and its antecedents in the local government context. Because of the positive potential outcomes of IWB, a number of companies have been interested in the invigoration of IWB in the workplace (Shanker et al., 2017; Saether, 2019). Under the difficult situations that local governments are in now, such as fiscal crises, scarce resources, demographic changes, and growing citizen expectations, IWB, as individual-level innovation, has been in the limelight in the public sector (Sørensen and Torfing, 2011; Bysted and Hansen, 2015; Miao et al., 2018). Korean local government employees have realized the importance of IWB as local governments have implemented various policies and programs to foster innovation and proactive behavior but local government employees are still regarded as being resistant to engaging in IWB due to job security, hierarchical culture, and avoidance of responsibility (Lee and Choi, 2016; Kim, 2017). In order to change their passive attitudes into IWB, the role of supervisors is critical (Hansen and Pihl-Thingvad, 2019; Khan and Khan, 2019). This study is to explore the role of supervisors in motivating their subordinates’ change-oriented behavior and positive attitudes toward their organization in the local government context.

## Theoretical Background and Hypotheses

### Supervisor Feedback and IWB

Supervisor feedback refers to “the extent to which supervisors provide their subordinates with valuable or helpful information that enables employees to develop, learn, adjust, and make improvements on the job” (Zhou, 2003, p. 415). Feedback is known as one of the most potent elements of behavior change or modification (Prue and Fairbank, 1981; Pinder, 2008). Pinder (2008) argues that feedback is one of the most inexpensive and easiest ways of changing behavior. Employees receive feedback from various sources: (a) supervisors or managers, (b) coworkers, (c) clients or customers, (d) self-generated, and (e) experts. According to Alvero's et al. (2001) review of the effectiveness of feedback, most previous studies found that supervisor feedback (86%) showed consistent effects on outcomes of feedback (e.g., individual performance and group performance). Among various sources of feedback, this study considers supervisor feedback in predicting desirable employee outcomes.

Expectancy theory suggests that feedback increases employees’ behavioral, attitudinal, and performance outcomes. People are motivated to behave in certain ways dependent on the belief that drives people to achieve specific outcomes (Vroom, 1964; Rosen et al., 2006). Feedback has characteristics as a motivational function that provides incentives and reinforces specific behaviors (Vroom, 1964; Ilgen et al., 1979). For example, Nelson (2013) contends that the feedback employees receive is one of the motivational forces that drive employees to have IWB and improve their performance. As employees receive feedback from their supervisors, they are more likely to have IWB to improve their performance.

One predominant theory that emphasizes the importance of perceived organizational support (POS) through feedback from supervisors is OST. According to OST, in order to meet the socio-emotional demands, “employees develop global beliefs and perceptions of organizational support concerning the extent to which the organization cares about their well-being and values their contribution” (POS; Eisenberger et al., 1986, p. 501). POS depends on employee’s attributions regarding their receipt of favorable or unfavorable treatment by their organization. OST posits that employees trade work effort and loyalty to the organization for social resources and tangible benefits. POS would increase the employees’ expectancy that their organization would reward them when they meet organizational goals and improve performance (Eisenberger et al., 1986; Kurtessis et al., 2017). Thus, employees who perceive organizational support are more likely to increase work effort and be obligated to reciprocate toward their organization. According to OST, employees consider supervisor support as a representative action on behalf of their organization (Jin and McDonald, 2017). Rhoades and Eisenberger (2002) found that supervisor support is strongly related to the employee’s perception of support. Supervisor feedback is one of the important components of supervisor support. Following the logic of OST, when employees receive feedback from their supervisors, they are more inclined to perceive considerable organizational support from their organization. In turn, this encourages employees to meet the organizational goals or improve their performance by displaying IWB, because the employees are willing to repay their supervisors’ favor by demonstrating positive work behaviors or attitudes (Eisenberger et al., 1986; Rhoades and Eisenberger, 2002; Eisenberger and Stinglhamber, 2011; Jin and McDonald, 2017).

Supervisor feedback affects IWB by clarifying goals and roles, providing work-relevant information and communicating organization’s values. First, supervisor feedback is a form of managerial intervention for decreasing uncertainty and clarifying roles and goals in an organization (Moynihan and Pandey, 2007; Lee, 2019). As supervisors succeed in clarifying goals and roles through feedback, subordinates are likely to better understand which tasks are critical to achieving goals. Benefits of providing feedback are to help focus attention on what employees have to do to achieve their own and organizational goals (Moynihan et al., 2012, p. 148). In addition, employees with goal clarity through supervisor feedback will have greater work motivation (Moynihan and Pandey, 2007). Whitaker and Levy (2012) argued that enhanced goal or role clarity as a result of feedback increases the extra-role and prosocial behaviors.

Second, supervisor feedback helps employees engage in IWB by providing work-relevant information and values and provides employees with the work-relevant information that enables them to learn, develop, and progress on the job (Zhou, 2003; Joo and Park, 2010). When supervisors provide employees with work-relevant information, employees acquire more knowledge and skills. As employees’ work-relevant knowledge and skills are crucial parts of individual creative self-efficacy, employees are more likely to generate novel ideas and use innovative skills at work through supervisor feedback (Su et al., 2019).

Third, when supervisors, as a representative of the organization, communicate the organization’s values through feedback to employees, employees are more likely to understand their organization’s key values. In addition, as employees understand their organization’s values, they are more likely to exhibit prosocial behaviors (Peng and Chiu, 2010). Especially, most Korean local governments’ key values are improving effectiveness, efficiency, and service quality through innovation and performance-oriented reform (Campbell, 2018; Nam, 2019). Therefore, when Korean local government employees understand that innovation and performance are key organizational values through supervisor feedback, they are more likely to choose to engage in IWB in order to realize those values in their own work. Previous studies have shown that vision, missions, and values that supervisors clearly articulate and communicate lead to employees’ prosocial and extra-role behaviors (Bottomley et al., 2016; Hansen and Pihl-Thingvad, 2019). Based on the theories and empirical evidence, it is hypothesized as follows:

*Hypothesis* 1: Supervisor feedback is positively related to IWB.

### The Mediating Role of Trust in Supervisor

Trust in supervisor has received considerable attention from researchers and practitioners regarding employees’ behaviors, work outcomes, and motivation. Trust in supervisor refers to the level of trust toward their supervisor that leads to positive outcomes within organizations (e.g., affective commitment, job performance, and OCB) based on an individual’s belief or confidence that their supervisor is competent, open, reliable, and helpful in an uncertain or risky situation (Mishra, 1996; Colquitt et al., 2007; Xiong et al., 2016). According to OST, supervisors can be viewed as a face-to-face representative of the organization because employees experience their organization directly through the supervisor’s actions, directions, and decisions. The development of trust between a trustee and a trustor has characteristics of reciprocity, in that individuals trust someone who offers growth possibilities, reduces uncertainty, provides useful resources, and gives information about performance (Carnevale and Wechsler, 1992). In addition, the norm of reciprocity posits that employees who perceive supervisor support through feedback are more obligated to reciprocate toward their supervisors (Rhoades and Eisenberger, 2002; Jin and McDonald, 2017). Feedback from supervisors may act as a vehicle for the building of trust between employees and supervisors in an organization (Peterson and Behfar, 2003).

The sense of trust in supervisor is positively associated with the development of IWB among public employees. Although several researchers contend that public employees working in a pervasive hierarchical culture are prone to avoid unknown risks (risk aversion) and are likely to work with guidance and clear rules (Hofstede et al., 2010; Kim, 2017), previous studies have suggested that trust in supervisors or leaders is an important proximal predictor of risk-taking behaviors, such as IWB (Dirks and Ferrin, 2002; Colquitt et al., 2007). Risk refers to “the extent to which there is uncertainty about whether potentially significant and/or disappointing outcomes of a decision will be realized” (Sitkin and Pablo, 1992, p. 10). Researchers have suggested that IWB can be risky because IWB has uncertainty and unpredictability over the outcomes and there could be the aftermath of the failure of IWB (Sitkin and Pablo, 1992; Shanker et al., 2017; Miao et al., 2018). However, trust in supervisor plays an important role in motivating employees to involve in risk-taking. In other words, when employees trust their supervisors, they are more likely to perform IWBs because they believe that their supervisors are reliable or competent enough to back them up in a risky situation. In addition, the risk-taking behavior (i.e., IWB) is affected by a contextual factor (Rodrigues and Veloso, 2013). Employees are more likely to attempt IWB in an organization where employees trust in their supervisors. Thus far, the author has contended that supervisor feedback leads to trust in supervisor, which in turn, contributes to IWB. Thus, it is hypothesized as follows:

*Hypothesis* 2: Supervisor feedback is positively related to trust in supervisor.
*Hypothesis* 3: Trust in supervisor is positively related to IWB.
*Hypothesis* 4: Trust in supervisor mediates the relationship between supervisor feedback and IWB.

### The Mediating Role of Affective Commitment

OC has drawn much attention among human resource development researchers. Researchers argue that the high level of OC is beneficial for both employees and organizations because the high level of OC provides intrinsic and extrinsic motivation. OC can play a role in security, identity, and comfort in an organization (Meyer and Allen, 1991; Pinder, 2008). In addition, previous studies have shown that OC is positively related to OCB, job performance, job involvement, job satisfaction, absenteeism, and turnover intentions (Nyhan, 1994; Meyer et al., 2002; Moynihan and Pandey, 2007; Joo and Park, 2010; Jin et al., 2018). After Allen and Meyer (1990) tested aspects of a three-dimensional model of OC, OC is viewed as a multi-dimensional construct: affective, normative, and continuance commitment. Researchers argue that affective commitment is the most vital explanatory variable among the three components because affective commitment has been more closely and significantly related to outcome variables (e.g., job satisfaction, performance, and creativity) than the other two components (Eby et al., 1999; Vandenberghe and Bentein, 2009). For this reason, a number of researchers have viewed affective commitment as representative of OC (De Witte and Buitendach, 2005, p. 29).

Affective commitment is defined as “an affective or emotional attachment to the organization such that the strongly committed individual identifies, is involved in and enjoys membership in the organization” (Allen and Meyer, 1990, p. 2). Feedback has been identified as an antecedent to affective commitment. Receiving feedback with competence and reliability from supervisors leads to the development of affective commitment among subordinates. For example, Joo and Park (2010) contend that developmental feedback from supervisors leads to greater affective commitment because when supervisors give employees behaviorally relevant information (i.e., feedback); the feedback helps employees to have positive psychological states toward their supervisors and organizations.

The relationship between feedback and affective commitment can be understood using social exchange theory. The concept of reciprocation by Levinson (1965) suggests that employees view their supervisor’s action as representative of the organization itself. Thus, the concept of reciprocation is by itself a vital driving force for creating and motivating an employee’s behavior for the sustainability of their organization (Eisenberger et al., 1986; Casimir et al., 2014; Tetteh et al., 2019). When a supervisor gives employees performance feedback, employees perceive the feedback as representative of their organization’s concern and support for their development, contributions, and well-being. Thus, feedback would increase affective commitment by exchanging loyalty and affective attachment for the organizational support they perceive (Hutchison and Garstka, 1996; Janssen, 2004; Eisenberger and Stinglhamber, 2011).

Affective attachment to the organization is positively related to the adoption or development of IWB. Researchers contend that employees who are affectively or emotionally committed to the organization are increasingly engaged in their organization and are willing to pursue organization’s goals and core values (Meyer and Allen, 1991; Gong et al., 2009; Camelo-Ordaz et al., 2011; Brimhall, 2019). In addition, highly affectively committed employees are more likely to take risks and thrive on challenges to be helpful to the organization than those who have low levels of affective commitment. Today, a number of organizations set a vision and goals for improving organizational performance and effectiveness by facilitating employees’ IWB and creativity. In order to thrive, employees’ IWB is required for organizations to cope with the difficulties and progressive changes in tumultuous times. Accordingly, employees with high levels of affective commitment are more inclined to perform IWB to achieve the organization’s goals and increase overall performance for the organization’s sustainable development. Although there is a lack of empirical research on the relationship between affective commitment and extra-role behavior (i.e., IWB), some empirical studies have suggested that they are linked. In a study of employee’s behavioral outcomes among Australian nurses, Xerri and Brunetto (2013) suggest that affective commitment is an important predictor of IWB because employees who are affectively committed to the organization are willing to improve organizational outcomes by displaying IWB. Olkkonen and Lipponen (2006, p. 207–208) found that organizational identification (i.e., the perception of oneness with or belongingness to the organization) is positively associated with extra-role behavior toward the organization (e.g., providing suggestions to improve their organization). Brimhall (2019) suggested that employees with high levels of affective commitment are more likely to increase perceptions of innovation because they are more willing to share ideas and increase social interaction with organizational members.

Drawing from social exchange theory, when employees receive performance/developmental feedback from supervisors, they are willing to devote more effort to the organization as in reciprocation for organizational support. This, in turn, encourages employees to have IWB. Given the theoretical and empirical evidence, it is posited that feedback from supervisors about employees’ performance enables employees to show higher affective commitment. This, in turn, fosters employee’s IWB through learning new methods, processes, and techniques and generating creative solutions to problems. Thus, it is hypothesized as follows:

*Hypothesis* 5: Feedback from supervisor is positively related to affective commitment.
*Hypothesis* 6: Affective commitment is positively related to IWB.
*Hypothesis* 7: Affective commitment mediates the relationship between feedback from supervisor and IWB.

**Figure 1:**
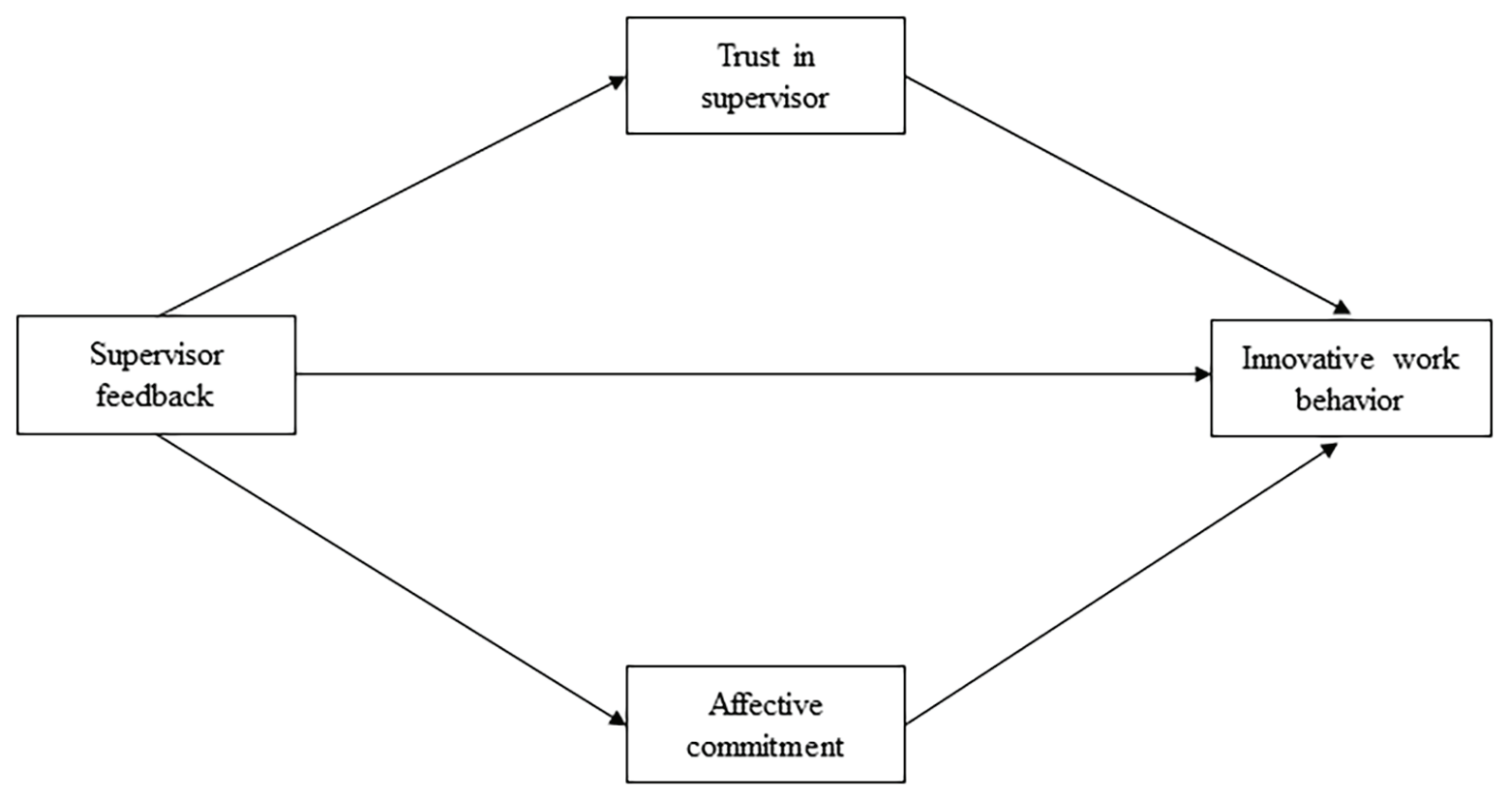
Hypothesized model.

The theorized model represented by the hypotheses is summarized in Figure 1.

## Materials and Methods

### Sample

The target population of this study is local government employees in South Korea. Firefighters, police officers, public school teachers, revenue officers, and public transportation workers were excluded from the target population. Public servants engaged in general service in local governments were included. Using a multistage cluster sampling method, the data used in this study were collected from 65 local governments, including provinces, metropolitan cities, cities, counties, districts, towns, townships, and neighborhoods government offices, in South Korea. The questionnaire was revised through pre-testing and piloting before initiating the survey. The questionnaire was administered from January 1, 2017 through February 14, 2017. Group-administered and self-administered questionnaires were used as data collection techniques. The questionnaires were distributed by the researcher and trained proctors. The participants were asked to complete a questionnaire during working hours, and retribution of the completed questionnaires was done after 2 or 3 h to maximize response rates. Participants were informed about the nature of the survey and of their right to decline participation. In addition, purposes of the study, assurances of confidentiality of data, and personal anonymity were explained by the researcher and trained proctors. This information was also written in the cover letter.

The questionnaires were distributed to 2,100 local government employees. A total of 1,724 questionnaires were returned, yielding an overall response rate of 82.1%. However, unreliable responses of 55 surveys were excluded, and a total of valid 1,669 responses were used for the analysis of the current study. Of the total respondents, 52.4% were men and 47.6% were women. In age, 8.5% were aged 20–29 years, 36.8% were aged 30–39, 32.2% were aged 40–49, and 22.5% were aged 50–60. In education, 82.8% had at least a bachelor’s degree, while 7.2% only had a high school diploma. Most (44.1%) of the respondents had served in the civil service for between 1 and 10 years, and the next-largest group was between 11 and 20 years of service.

### Measures

This study used measures that have been validated in the literature. All items, except demographic factors, were measured using a seven-point Likert scale ranging from 1 (strongly disagree) to 7 (strongly agree). The questionnaire was originally developed in English and then translated into Korean to increase participants’ understanding. In order to ensure accuracy and equivalency of the two versions of the questionnaire, forward and back translation techniques were applied.

### Supervisor Feedback

Supervisor feedback was measured using five items adapted from the feedback environment scale (FES) developed by Steelman et al. (2004). They developed and validated the scale to understand more about the feedback process and the nature/facets of the feedback environment in organizations (Steelman et al., 2004; Rosen et al., 2006). Representative items are “My supervisor gives me useful feedback about my job performance” and “When I do a good job at work, my supervisor praises my performance.” The Cronbach’s alpha for feedback was 0.92.

### Trust in Supervisor

Trust in supervisor was measured with four items using the trust instrument (TI) developed by Nyhan and Marlowe (1997). Nyhan and Marlowe (1997) developed the TI based on the employee perception questionnaire (EPQ) and the organizational trust inventory (OTI) to assess the individual’s level of trust in their supervisor (Nyhan, 1994; Nyhan and Marlowe, 1997). Participants were asked to rate the degree to which they trust their supervisors. Sample items include “I have confidence that my supervisor is technically competent at the critical elements of his/her job” and “My supervisor will back me up in a pinch.” Cronbach’s alpha for trust in supervisor was 0.93.

### Affective Commitment

Affective commitment was measured with six items from Allen and Meyer’s (1990) three-component model of commitment. This instrument has been widely used to assess affective commitment because the affective commitment scale has shown good reliability and validity (Allen and Meyer, 1996; De Witte and Buitendach, 2005). Participants were asked to rate the level of “their affective or emotional attachment to, identification with, and involvement in the organization they work for” (Meyer and Allen, 1991, p. 67). Sample items include “I really feel as if my organization’s problems are my own” and “The organization has a great deal of personal meaning to me.” Cronbach’s alpha for affective commitment was 0.92.

### Innovative Work Behavior

IWB was measured with five of six items from Scott and Bruce’s (1994) innovative behavior measure and one item from Janssen’s (2000) study. Participants were asked to rate the extent to which they express their willingness to seek ways to generate, promote, implement, and realize new ideas (Scott and Bruce, 1994; Janssen, 2000). Representative items are “I try to generate creative solutions to problems,” “I promote and champion idea to other,” “I develop adequate plans and schedules for the implementation of new ideas,” and “I try to secure the funding and resources needed to implement innovations.” Cronbach’s alpha for IWB was 0.94.

### Control Variables

The author controlled for confounding variables that correlate with both the independent variable and the dependent variable. Therefore, we controlled for demographic variables, such as gender (0 = male, 1 = female), educational level (1 = less than high school, 2 = high school graduate, 3 = some college, 4 = bachelor’s degree, 5 = master’s degree, and 6 = doctorate degree), marital status (0 = single, 1 = married), age (1 = 20–29, 2 = 30–39, 3 = 40–49, 4 = over 50), and tenure (1 = less than 5 years, 2 = 6–10 years, 3 = 11–15 years, 4 = 16–20 years, 5 = 21–25 years, and 6 = over 26 years).

## Results

### Test of Common Method Bias

Common method bias is a problem “which threatens the validity of the conclusions about the relationships between measures” when conducting a self-administered survey at the same point in time (Podsakoff et al., 2003, p. 879). To address the potential problem for common method bias, this study conducted two tests: Harman’s single-factor test and confirmatory factor analysis (CFA). First, a Harman’s single-factor test was conducted to see whether a single factor accounts for a majority of the covariance in the data (Podsakoff et al., 2003; Jin and McDonald, 2017). All items from each of the constructs entered into an exploratory factor analysis with varimax rotation. The results of the principal component analysis showed that the eigenvalues of the four factors were greater than 1.0. In addition, the four factors together accounted for 80% of the total variance, whereas the largest factor accounted for only 45% of the variance, lower than the cut-off value of 50%. Second, the results of the CFA showed that the single-factor model had a poor fit, with *χ* ^2^(153) = 13652.14, GFI = 0.440, CFI = 0.548, NFI = 0.546, IFI = 0.549, and RMSEA = 0.230. The results of the two analyses show that data in this study are unlikely to have the possibility of common method bias.

### Measurement Model

Table 1 presents the correlations, means, standard deviations, and average variance extracted (AVE) for the measures used in this study. All correlation coefficients were statistically significant (*p* < 0.01). The construct reliability (CR), convergent validity, and discriminant validity of the measurement model were assessed by the CFA. All CR values were greater than 0.7. In terms of convergent validity, factor loadings of all items were over 0.7, and in a range between 0.771 and 0.937. All AVE values were over 0.5. In terms of discriminant validity, the square root of AVE of each construct was larger than the inter-construct correlations in the model. In addition, the interval of confidence of the correlation of variables in the model did not contain one. In sum, the results show that the instrument in the present study has reliability, convergent validity, and discriminant validity.

**Table 1:** Descriptive statistics, correlations, and average variance extracted (AVE).

Variables	1	2	3	4	5	6	7	8	Mean	SD	AVE
1 Gender									0.48	0.50	
2 Age	−0.29^**^								4.73	1.31	
3 Marital status	−0.22^**^	0.57^**^							0.71	0.45	
4 Education	0.08^**^	−0.15^**^	−0.05^*^						3.84	0.68	
5 Tenure	−0.19^**^	0.86^**^	0.53^**^	−18^**^					3.47	1.96	
6 Supervisor feedback	−0.05^*^	−0.05^*^	0.02	0.01	−0.04				5.21	1.03	0.69
7 Trust in supervisor	−0.04	0.12^**^	0.08^**^	0.03	0.11^**^	0.41^**^			4.60	1.02	0.73
8 Affective commitment	−0.05	0.19^**^	0.12^**^	0.01	0.18^**^	0.40^**^	0.68^**^		4.70	1.03	0.68
9 Innovative work behavior	−0.11^**^	0.20^**^	0.18^**^	0.08^**^	0.18^**^	0.35^**^	0.56^**^	0.49^**^	4.55	0.97	0.71

* *p* < 0.05;

** *p* < 0.01;

*n* = 1669.

The goodness-of-fit indices of the measurement model show how well it fits a set of observations. As shown in Table 2, the measurement model with four factors provided better fit to the data (GFI = 0.923, CFI = 0.964, TLI = 0.958, IFI = 0.964, and RMSEA = 0.067) than other alternative models. According to the result of the CFA for constructs, the measurement model showed good model fit and, therefore, construct validity was guaranteed.

**Table 2:** Assessment of the measurement model.

	*χ* ^2^	*df*	CFI	GFI	IFI	TLI	RMSEA
Four-factor model (FB; TS; AC; IWB)	1228.722	146	0.964	0.923	0.964	0.958	0.067
Three-factor model (FB; TS + AC; IWB)	4344.542	149	0.860	0.729	0.860	0.839	0.130
Two-factor model (FB + TS +AC; WB)	6831.657	151	0.777	0.648	0.777	0.747	0.163
Two-factor model (FB; S +AC + IWB)	11249.301	152	0.629	0.471	0.629	0.582	0.209
Single-factor model (FB + TS +AC + IWB)	13652.135	153	0.548	0.440	0.549	0.495	0.230

FB, supervisor feedback; TS, trust in supervisor; AC, affective commitment; IWB, innovative work behavior.

### Hypothesis Testing

**Figure 2:**
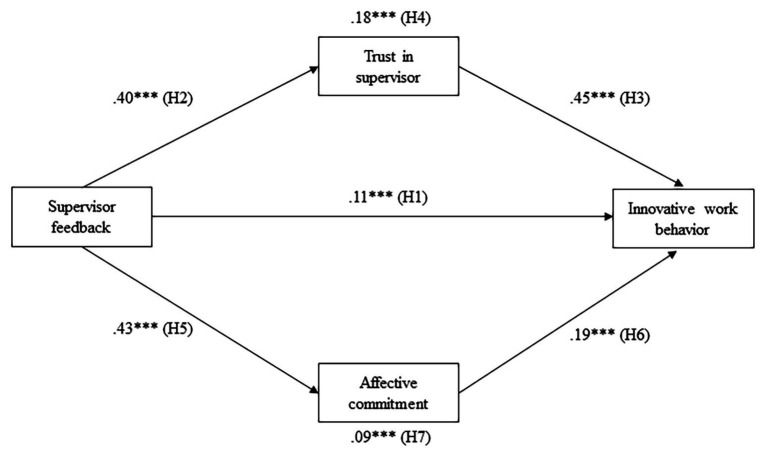
Standardized path coefficients for the mediation model. ^***^ *p* < 0.001.

Hypotheses were tested using structural equation modeling (SEM) with the AMOS 22.0. The standardized path coefficients are summarized in Figure 2.

As predicted in Hypothesis 1, feedback from supervisor was found to have a significant effect on IWB (*β* = 0.11, *p* < 0.001). Feedback from supervisor was also found to be positively related to trust in supervisor (*β* = 0.40, *p* < 0.001) and affective commitment (*β* = 0.43, *p* < 0.001), thereby supporting Hypotheses 2 and 5. Trust in supervisor and affective commitment were found to have a positive effect on IWB (*β* = 0.45, *p* < 0.001; *β* = 0.19, *p* < 0.001, respectively); hence, Hypotheses 3 and 6 were supported.

To test the mediation hypotheses and the significance of indirect effects, this study employed a bootstrapping test at 95% bias-corrected bootstrap confidence intervals with 10,000 samples. When the 95% bias-corrected bootstrap confidence intervals do not contain zero, the mediation effect can be interpreted as statistically significant. The results of a bootstrapping test are presented in Table 3. The results showed that trust in supervisor significantly mediated the relationship between feedback from supervisor and IWB, and the indirect effect was 0.18 (*p* < 0.001). Affective commitment was also found to mediate the relationship between feedback from supervisor and IWB, and the indirect effect was 0.09 (*p* < 0.001). Furthermore, all indirect effects were statistically significant as any bootstrap confidence interval does not contain zero; thus, Hypotheses 4 and 7 were supported. Therefore, the results provided evidence of the proposed parallel mediation model.

**Table 3:** Bootstrapping results for indirect effects.

Path	Estimate	Bias-corrected bootstrap (95% CI)
Supervisor feedback→Trust in supervisor→IWB	0.18^***^	(0.1684, 0.2253)
Supervisor feedback→Affective commitment→IWB	0.09^***^	(0.1396, 0.1922)

*** *p* < 0.001.

Bootstrap confidence intervals were constructed using 10,000 resamples. IWB, innovative work behavior.

## Discussion

IWB and its determinants, supervisor feedback, trust in supervisor, and affective commitment, have gained an increased interest in the public sector. Because of the relatively few studies of the mechanism through which supervisor feedback influences IWB, several unaddressed questions still remain. Drawing from OST and social exchange theory, this study proposed and tested a mediation model to remove the veil about a mechanism of supervisor feedback on IWB that includes two mediators, trust in supervisor and affective commitment, among Korean local government employees.

### Theoretical Implications

Four theoretical contributions emerge from this study. First, drawing from OST, this study demonstrated that supervisor feedback has a positive direct effect on IWB. Although human resource development researchers argued that supervisor feedback is linked to behavioral and attitudinal outcomes, the relationship between supervisor feedback and IWB has rarely been explored to any significant degree. In addition, previous studies viewed feedback as a moderator in the antecedents-IWB relationship, with various samples including public university employees in Italy (Battistelli et al., 2013) and employees at a high-tech company in Germany (Schaffer et al., 2012). These previous studies found that feedback moderated the relationship between IWB and its antecedents, whereby the relationship was stronger when employees received high levels of feedback. By demonstrating the direct effect of supervisor feedback on IWB, this study contributes to the emerging literature that focuses on the critical role of feedback as an important antecedent of IWB.

Second, this study found that Korean local government employees who receive performance/developmental feedback from their supervisors develop trust in their supervisors, which consequently leads to IWB. The model tested suggests that trust in supervisor plays a significant role in mediating the relationship between supervisor feedback and IWB in the Korean context. This study also demonstrated the importance of trust in the IWB development process in an organization. It has been known that trust in supervisors associated with feedback leads to positive outcomes, such as productivity (Nyhan, 2000), OC (Nyhan, 2000), and performance (Earley, 1986; Favero et al., 2014), whereas IWB has not been thoroughly explored regarding the mechanism of feedback to trust. As per the results of Hypothesis 4, IWB is a possible outcome from the mechanism of feedback to trust. In addition, previous studies found that supervisor feedback has an indirect, positive effect on performance through its influence on trust (Earley, 1986; Favero et al., 2014) and that IWB is positively associated with performance in government (De Jong and Den Hartog, 2008; Fernandez and Moldogaziev, 2013). Fernandez and Moldogaziev’s (2013) study using the Federal Human Capital Survey (FHCS) among U.S. government employees suggests that the effect of innovative behavior on performance in the public sector is positive in the long term. Thus, we may add performance to this model and postulate that feedback has an indirect effect on performance through its effect on trust and IWB as a causal chain. This could open a new chapter for IWB-performance literature.

Third, this study found that the relationship between supervisor feedback and IWB is mediated by affective commitment. According to previous studies, when employees receive feedback from their supervisors, which reduces uncertainty in the workplace and supports subordinates to perform better, they are emotionally attached to their organization. Employees perceive feedback as valuable organizational support and feel that their organization is concerned about their well-being. These feelings, in turn, motivate employees to exhibit affective commitment to the organization (Hutchison and Garstka, 1996; Joo and Park, 2010). In addition, employees who are emotionally attached to their organization are more likely to have IWB (Farnese et al., 2016). Previous studies have dealt with affective commitment as a dependent variable or a possible outcome associated with feedback (Nyhan, 1994, 2000; Hutchison and Garstka, 1996). There were not many attempts to use IWB as an outcome of how feedback leads to affective commitment. In addition, the mechanism of the relationship between feedback and IWB through its influence on affective commitment has received little attention in either the private or the public sector. However, the findings of this study demonstrated the importance of affective commitment in the relationship between feedback and IWB, and the mechanism of feedback to affective commitment can be expanded to IWB. Thus, this study contributes to the expansion of literature that reveals the critical role of affective commitment as a motivating mechanism of the relationship between feedback and IWB.

### Practical Implications

This study provides practical implications for supervisors, practitioners, and human resource managers. First, the results of this study suggest that supervisor feedback is ineffective when it is used alone; however, it is significantly related to IWB through its effect on trust in supervisor and affective commitment, which in turn affect IWB. Following the logic of OST, supervisors in local governments should provide feedback to subordinates with care and concern that makes them feel more obligated to reciprocate with greater IWB. In addition, feedback delivery is effective when supervisors give feedback with the candidness that they believe is helpful for recipients’ (i.e., subordinates) development, motivation, and success. Effective feedback delivery encourages subordinates to exhibit greater trust in supervisors and to become emotionally attached to the organization. In addition, supervisors should give subordinates feedback that enables them to achieve better performance. If the feedback does not help them to improve their performance, they may express doubt about the usefulness of the feedback. According to London (2015), feedback delivery changes the focus of attention and results in behavior change. If the feedback supervisors deliver is not effective for performance improvement, subordinates may not trust their supervisors. In turn, this makes it difficult for them to exhibit IWB because of poor feedback, which cannot lead to performance improvement, making it hard to change the behaviors among subordinates (London, 2015). As a result, local governments need to: (a) realize the importance of feedback as a bridgehead for building trust in supervisors, affective commitment, and IWB, (b) train supervisors to learn how to provide feedback effectively, and (c) encourage supervisors to use various skills of feedback delivery (e.g., clear and concise communication, and timely and regular feedback).

The findings of this study show that positive feedback from supervisors leads to greater affective commitment, hence more IWB because of reciprocation toward the organization. Therefore, supervisors and managers should consider delivering feedback in the form of compliments. Nelson and Schunn (2009) suggest that feedback in the form of praise increases one’s agreement with another following feedback and a better understanding of that feedback. In addition, when employees receive feedback in the form of praise at the right time, they are more inclined to perform better, put more effort into their job, and become emotionally attached to their organization (Nelson and Schunn, 2009). It is also important to provide feedback relating to the organization’s visions, goals, and values. Norris-Watts and Levy (2004) found that if supervisors share the organization’s goals and values through feedback, employees are more likely to show affective commitment. This, in turn, leads to behavior changes for individual and organizational development. These behavioral changes are often done by IWB, such as the application of new ideas, task revision, and correction of a faulty procedure (Crant, 2000).

According to feedback from survey respondents, unimportant feedback is often delivered to solve a minor problem at work. When employees solve the problem with supervisor feedback, they are more likely to trust their supervisors. However, this kind of feedback has characteristics that pass on know-how for the minor problem but may not enable their subordinates to generate creative solutions to problems. Although this adaptation to circumstances in problem-solving with the feedback may be helpful to their performance in the short term, these tactics appear to have a negative effect on developing employee’s IWB. In order to promote IWB among employees, supervisors should deliver feedback that enables subordinates to (1) identify the causes of problems, (2) learn the responsibilities and purposes of work they do, (3) develop their own problem-solving ability, and (4) become more aware of the ultimate goals and values of their organization for developing IWB in the long term.

According to OST, supervisors are viewed as agents who act on behalf of the organization (Jin and McDonald, 2017). Thus, trust, affective commitment, and IWB within the organization depend on the quality of the subordinate-supervisor relationship. It is vital to create a work climate where supervisors deliver feedback frequently and authentically with an understanding of subordinates’ needs and demands in mind. In addition, public organizations should invest in creating a culture where open communication is active between supervisors and subordinates. In Korea, communication between supervisors and subordinates has not been highly developed because of the hierarchical culture derived from Confucianism, where subordinates follow their supervisor’s directions and instructions. This strict and uncomfortable communication atmosphere hampers feedback exchange and creative idea exchange in an organization. Earlier studies of the Korean government culture found that Korean public employees working in hierarchical cultures are less likely to be affectively committed to the organization where it is difficult to express their opinions (Kim, 2012; Park et al., 2013). In addition, Korean local government employees working in strict cultures are less likely to communicate with their supervisors. This strict top-down culture may not foster trust in supervisor and affective commitment (Lee, 2008). Therefore, it is suggested that Korean local governments should create an organizational culture in which free communication, cooperation, and frequent feedback between subordinates and supervisors are invigorated. It is expected that this organizational culture would improve trust, affective commitment, and IWB among Korean local government employees.

### Limitations and Future Research

The findings of this study should be interpreted with caution because this study has some limitations. First, the use of cross-sectional data may not allow us to draw causality between the variables used in this study. Although the author used several procedural and statistical techniques to address the causality issue regarding the use of cross-sectional data, future research should use longitudinal data (e.g., panel or pooled cross-sectional data) to confirm the causal order among variables. Second, this study was conducted using self-reported data. The use of self-reported data may raise the possibility of the social desirability bias or common method bias. These biases may inflate or deflate relationships between variables used in this study. Although the author included survey questions regarding social desirability bias and conducted a pilot test and a pre-test to reduce the possibility of the biases, the author may not completely be able to discount them. Some researchers suggested that self-reports are useful when measuring individuals’ perceptions, feelings, beliefs, and judgment and can be more subtle than the manager or supervisor rates when it comes to measuring individual performance-related outcomes (Janssen, 2001; George and Pandey, 2017). However, using both self-reported and supervisors’ or managers’ ratings of individual performance-related outcomes should be considered in future research. Third, the author needs to recognize the lack of generalizability, in that the findings may not be applicable to other groups or populations. Data were collected from various local government types to guarantee the representativeness of the target population. However, the characteristics and culture of local governments may differ depending on their location, size, demographics, budget, resources, and scope of services (Jin et al., 2018).

It is necessary to discuss why the feedback-IWB relationship in the Korean local government context is distinct from that in other contexts, such as private firms or local governments in Western countries or other Asia Pacific countries. The mechanisms of supervisor feedback on IWB could be explained by the coexistence of collectivism and individualism in Korean local governments. It is known that that Korean local governments’ organizational culture is based on collectivism from Confucianism (Kim, 2017). However, because of rapid economic development, urbanization, and increased opportunities for higher education, individuals are becoming more individualistic and taking actions to optimize their utility and well-being (Bak, 2019). Respondents of this study reported that they receive a considerable amount of performance feedback from their supervisors in general, which is likely to have resulted from the collectivistic culture that focuses on organizational development. Because of this, people are more likely to behave in a way that is beneficial to their own organizations. With this motive, supervisors are willing to give subordinates performance feedback for organizational members’ development. In turn, employees who receive performance feedback from their supervisors also increase trust and affective commitment for organizational development affected by the collectivistic culture. Researchers suggest that collectivism and individualism have different effects on individual attitudes and outcomes, and organizational outcomes, such as public service motivation, OC, trust, OCB, and performance, among public servants (Park et al., 2013; Kim, 2017). However, this study shows a surprising result, in that Korean local government employees may behave by both collectivism (i.e., group interests) and individualism (i.e., self-interest). Thus, future research should examine the mechanisms of how conflicting cultural values, collectivism, and individualism interact with the feedback-IWB relationship. For example, future research can explore how those cultures influence supervisors’ feedback delivery behaviors and individuals’ engagement in IWB among both public and private sector organizations in other Asian countries, where collectivism and individualism coexist or may compete.

## Data Availability Statement

The datasets generated for this study are available on request to the corresponding author.

## Ethics Statement

This study involving human subjects was reviewed and approved by the Institutional Review Board (IRB) of Virginia Commonwealth University. Participation in this study was voluntary. Purposes of the study, assurances of confidentiality of data, and personal anonymity were informed by researchers and written in the cover letter.

## Author Contributions

HB designed the research model, supervised the data collection, analyzed the data, and wrote the manuscript.

### Conflict of Interest

The author declares that the research was conducted in the absence of any commercial or financial relationships that could be construed as a potential conflict of interest.
